# Electrophysiological and structural determinants of electrotonic modulation of repolarization by the activation sequence

**DOI:** 10.3389/fphys.2013.00281

**Published:** 2013-10-08

**Authors:** Richard D. Walton, Alan P. Benson, Matthew E. L. Hardy, Ed White, Olivier Bernus

**Affiliations:** ^1^Faculty of Biological Sciences, Multidisciplinary Cardiovascular Research Centre, School of Biomedical Sciences, Institute of Membrane and Systems Biology, University of LeedsLeeds, UK; ^2^Unité Inserm 1045, Centre de Recherche Cardio-Thoracique, Université Bordeaux SegalenBordeaux, France; ^3^L'Institut de Rythmologie et Modélisation Cardiaque, Université de BordeauxBordeaux, France

**Keywords:** action potential duration, heterogeneity, ventricular repolarization, electrotonic current, cardiac electrophysiology

## Abstract

Spatial dispersion of repolarization is known to play an important role in arrhythmogenesis. Electrotonic modulation of repolarization by the activation sequence has been observed in some species and tissue preparations, but to varying extents. Our study sought to determine the mechanisms underlying species- and tissue-dependent electrotonic modulation of repolarization in ventricles. Epi-fluorescence optical imaging of whole rat hearts and pig left ventricular wedges were used to assess epicardial spatial activation and repolarization characteristics. Experiments were supported by computer simulations using realistic geometries. Tight coupling between activation times (AT) and action potential duration (APD) were observed in rat experiments but not in pig. Linear correlation analysis found slopes of −1.03 ± 0.59 and −0.26 ± 0.13 for rat and pig, respectively (*p* < 0.0001). In rat, maximal dispersion of APD was 11.0 ± 3.1 ms but dispersion of repolarization time (RT) was relatively homogeneous (8.2 ± 2.7, *p* < 0.0001). However, in pig no such difference was observed between the dispersion of APD and RT (17.8 ± 6.1 vs. 17.7 ± 6.5, respectively). Localized elevations of APD (12.9 ± 8.3%) were identified at ventricular insertion sites of rat hearts both in experiments and simulations. Tissue geometry and action potential (AP) morphology contributed significantly to determining influence of electrotonic modulation. Simulations of a rat AP in a pig geometry decreased the slope of AT and APD relationships by 70.6% whereas slopes were increased by 75.0% when implementing a pig AP in a rat geometry. A modified pig AP, shortened to match the rat APD, showed little coupling between AT and APD with greatly reduced slope compared to the rat AP. Electrotonic modulation of repolarization by the activation sequence is especially pronounced in small hearts with murine-like APs. Tissue architecture and AP morphology play an important role in electrotonic modulation of repolarization.

## Introduction

Regional variations of the action potential duration (APD) are perhaps the best characterized form of electrophysiological heterogeneity in the heart. It has been reported in a wide range of species that spatial gradients of APD exist from base to apex and in the transmural plane of healthy myocardium (Wan et al., 2000; Antzelevitch, 2005). Such variations in APD serve to co-ordinate repolarization time (RT) of tissue to maintain normal functioning of the heart and are determined at a cellular level by the density of transmembrane ionic currents in various regions of the heart (Nerbonne and Kass, 2005).

At tissue level, intrinsic APD heterogeneity can be modulated by electrotonic interactions between cells (Laurita et al., 1996). Furthermore, such electrotonic interactions can lead to acute modulation of APD gradients depending on the activation sequence, leading to inverse linear relationships between activation time (AT) and APD (Franz et al., 1987; Laurita et al., 1997; Yuan et al., 2001; Banville and Gray, 2002; Yue et al., 2005; Chauhan et al., 2006; Hanson et al., 2009; Myles et al., 2010). The dynamic nature of this modulation has been attributed, in part, to the spatial gradient in membrane potential occurring during the repolarization phase of a propagating AP. Each cell is influenced by electrotonic load from its neighbors such that, cells repolarizing later generate an inward electrotonic current to their earlier repolarizing neighbors. In homogeneous tissue, this effectively prolongs the APD of the earlier activated cells and generates gradually decreasing APDs away from the pacing site. These APD gradients are the most pronounced at the pacing site, at the tissue boundaries and in directions of slow propagation (Zubair et al., 1994).

Several computational studies have investigated the effects of electrotonic currents on repolarization in cardiac tissue. Sampson and Henriquez (2005) for example, observed that electrotonic interactions could completely mask the intrinsic transmural APD gradient in the small mouse heart, but not in the larger rabbit heart. A more recent computational study by Cherry and Fenton (2011) found that boundaries act as a sink and shorten APD in otherwise homogeneous tissues. Moreover, APD was abbreviated where APs collide, or significantly increased where APs travel around sharp cusps in tissue geometry, such as occurring at the insertion site of the septum with the ventricular wall. In experiments, electrotonic modulation of repolarization by activation sequence has been observed to various extents in myocardium of different species, including humans, and various tissue preparations (Franz et al., 1987; Laurita et al., 1997; Yuan et al., 2001; Banville and Gray, 2002; Yue et al., 2005; Chauhan et al., 2006; Hanson et al., 2009; Walton et al., 2010). However, the species-to-species variability and the effects of tissue geometry have not yet been shown nor elucidated experimentally.

In the present study we aimed to determine the mechanisms underlying species- and tissue-dependent electrotonic modulation of repolarization in ventricles. Therefore, we investigated acute electrotonic APD modulation by activation sequence in two distinct species, the rat and the pig, and two different tissue preparations, the intact heart (rat) and the left ventricular wedge preparation (pig). The rat is used extensively in cardiac electrophysiology studies (Macchi et al., 2004; Rossi et al., 2008; Wasserstrom et al., 2009), while the pig is more closely related to human in terms of cardiac electrophysiology (Yuan et al., 2001). We used an epi-fluorescence optical mapping technique that provides high spatio-temporal resolution of electrical activity (Walton et al., 2010) to relate epicardial AT to APD and RT. We also utilized detailed computational models of electrical propagation in realistic tissue geometries obtained by diffusion tensor magnetic resonance imaging (DT-MRI) to investigate the relative roles of AP morphology and tissue geometry on electrotonic modulation of repolarization.

## Methods

### Tissue preparation

All experimental protocols conformed to the Animals (Scientific Procedures) Act 1986. Male Wistar rats (*N* = 8) weighing 220–250 g were euthanized by stunning and cervical dislocation and hearts rapidly excised. Hearts were submersed in cold (4°C) cardioplegic solution containing (in mmol/L): glucose, 277.5; KCl, 30; NaHCO_3_, 25; mannitol, 34.3, pH 7.4. The aorta was cannulated and perfused at 7 ml/min with bicarbonate buffered saline solution containing (mmol/L): NaCl, 130; NaHCO_3_, 24; NaH_2_PO_4_, 1.2; MgCl_2_, 1; glucose, 5.6; KCl, 4; CaCl_2_, 1.8; oxygenated with 95% O_2_/5% CO_2_, pH 7.4, 37°C. Female pigs (24–26 Kg, *N* = 9) were euthanized by intraperitoneal injection with sodium pentobarbital (35 mg/Kg) and the hearts were quickly excised. The aorta was cannulated and perfused with cold cardioplegic solution supplemented with heparin (5 U/ml). The left ventricular wall was dissected and the left anterior descending coronary artery was cannulated and perfused with bicarbonate buffered saline solution at 20 ml/min. In all experiments, the perfusate was supplemented with 10 μM blebbistatin for mechanical uncoupling of the myocardium.

### Optical mapping protocol and setup

The tissue was stained with potentiometric dye DI-4-ANEPPS (50 μg/ml bicarbonate buffered saline solution) via the perfusate at the beginning of the experiment (Walton et al., 2010). Bipolar electrodes were used to stimulate the ventricles over a range of basic cycle lengths from 160 to 83 ms for rats and 1000–256 ms for pigs. Optical recordings were acquired through a high-frame-rate charge-coupled device video camera (SciMeasure Analytical systems, GA, USA) mounted with a lens (focal length 12 mm, 1:0.8 aperture ratio; Computar, London, UK). Excitation light from monochromatic LEDs, 530 nm, (Cairn Research Ltd, Kent, UK) illuminated the epicardial surface. Emission light from DI-4-ANEPPS was filtered through a broadband 700DF50 filter. Images (80 × 80 pixels) with pixel dimensions of 0.25 × 0.25 mm for rats and 0.4 × 0.4 mm for pigs were acquired at 1000 frames per s. Background fluorescence was subtracted from each frame to obtain the voltage-dependent optical signal. Optical APs acquired over 10 s were ensemble averaged and underwent temporal (3 ms kernel) and spatial (1.25 mm kernel) filtration.

### Diffusion tensor MRI

We determined tissue geometry and architecture using DT MRI. Following optical mapping experiments, hearts, and ventricular wedge preparations were perfused with 4% formalin. Fixed hearts were immersed in the perfluoropolyether Fomblin (Sigma-Aldrich, St. Louis, USA) to reduce noise and enhance image contrast. High resolution (200 μm isotropic) imaging of the fiber orientation was performed with a 9.4 T NMR instrument (Bruker BioSpin, Ettlingen, Germany). Diffusion of protons was measured throughout the tissue in a set of 12 optimized directions (Papadakis et al., 1999) using a 3D diffusion-weighted spin-echo sequence with reduced encoding at 20°C (500 ms repetition time; 15 ms echo time; diffusion gradients with 2 ms duration and 7 ms separation; *b* = 1000 s mm^−2^). Diffusion tensors, and the eigenvectors and eigenvalues of these tensors, were calculated from the diffusion measurements at each voxel throughout the tissue using in-house software. No smoothing or interpolation of the diffusion measurements was necessary: see Benson et al. (2011b) for examples of the calculated fiber directions in rat hearts.

### Computer simulations

As in previous studies we used a monodomain approach to simulate AP wave propagation in cardiac tissue (Walton et al., 2010). Realistic geometry and fiber orientation (represented in the models by the electrical diffusion tensor **D**) were obtained from DT-MRI (see above):

**D** at a particular point in space is given by

$$\text{D}=D_{1}\text{I}+(D_{1}-D_{2}){\text{e}}_{1}{\text{e}}_{1}^{\text{T}}$$

where *D*_1_ and *D*_2_ are electrical diffusions along and across the fiber, respectively (both in mm^2^ms^−1^), **I** is the identity matrix, **e**_1_ is the primary eigenvector from DT-MRI, and the superscript T denotes vector transpose. We set *D*_1_ to 0.095 mm^2^ms^−1^ in the pig model and 0.17 mm^2^ms^−1^ in the rat model, which gave conduction velocities of 0.6 ms^−1^ in the fiber direction in both cases. For anisotropic propagation, we used a 1/4 diffusion ratio (i.e., *D*_2_ = *D*_1_/4) in rat and 1/9 diffusion ratio in pig, which gave conduction velocities of 0.3 and 0.15 ms^−1^, respectively, in the cross-fiber direction to match conduction velocity ratios measured by optical mapping. Electrical excitation at a particular point in the heart is then given by

$$\frac{\partial V}{\partial t}=\nabla (\text{D}\nabla V)-I_{\text{ion}}$$

where *V* is membrane potential (mV), *t* is time (ms), ∇ is a spatial gradient operator, and *I*_ion_ is total membrane ionic current density (μA/μ F). For *I*_ion_ in the rat, we used the model of Pandit et al. (2001) with constant ion concentrations, *Q*_10_ modifications for temperature as in Noujaim et al. (2007). In addition, *G*_Ca,L_ was increased by 75% and *G*_to_ decreased by 25% so that the model APD matched experimentally-recorded APDs. For *I*_ion_ in the pig, we used the Luo-Rudy dynamic guinea pig model (Faber and Rudy, 2000) (as no model for the pig is currently available), also with constant ion concentrations, and with *G*_Ca,L_ increased by 25% and *G*_Kr_ and *G*_Ks_ both decreased by 30%, so that the model APD matched experimentally-recorded pig APD. For a subset of simulations we increased *G*_Kr_ and *G*_Ks_ by a factor of 28.5 to match the pig APD to the rat APD. In the rat left ventricle a linear transmural gradient in selected ion channel densities was implemented as in Pandit et al. (2001), Walton et al. (2010). For rat right ventricular tissue, the epicardial model conductances were scaled according to the values in Benoist et al. (2011). For the pig model, a transmural gradient was introduced by assuming the default model (Faber and Rudy, 2000) was epicardial, increasing *G*_Na_ by 33% and reducing *G*_to_ by 64% for the endocardial model, then linearly scaling these parameters as a function of transmural distance. In all cases, pacing was via a twice-diastolic threshold stimulus current for 10 beats (500 and 167 ms basic cycle lengths for the pig model or rat model, respectively). The models were coded in C, and solved using a forward-time center-space method (Press et al., 2007) and an operator splitting and adaptive time step technique (Qu and Garfinkel, 1999), with a fixed space step of 0.2 mm (as determined by the DT-MRI resolution), a minimum time step of Δ *t*_min_ = 0.01 ms, and a maximum time step of Δ *T* = 0.25 ms. No-flux boundary conditions were imposed by setting electrical diffusion along the vector normal to the local tissue surface to zero, a standard approach for finite difference models. See (Benson et al., 2008, 2011a) for further details.

### Data analysis and statistics

AT was measured at the level of the maximal time derivative of the AP upstroke in experiments and simulations. APDs were measured from the AT to 80% of repolarization (APD_80_). RT was measured at the time of 80% of repolarization (RT = APD_80_ + AT). Linear correlation analysis was used to determine the relationship between AT and APD_80_ by comparing gradients of fits. Dispersion of APD_80_ and RT were measured as the difference between their respective 5 and 95% confidence intervals of normal sample distributions. Pixels incorporated in the measurement of dispersion were restricted to those with AT ≤10 ms. Statistical analyses using paired and unpaired *t*-tests determined significance when *p* < 0.05.

## Results

### The distribution of the action potential duration

**Figure 1:**
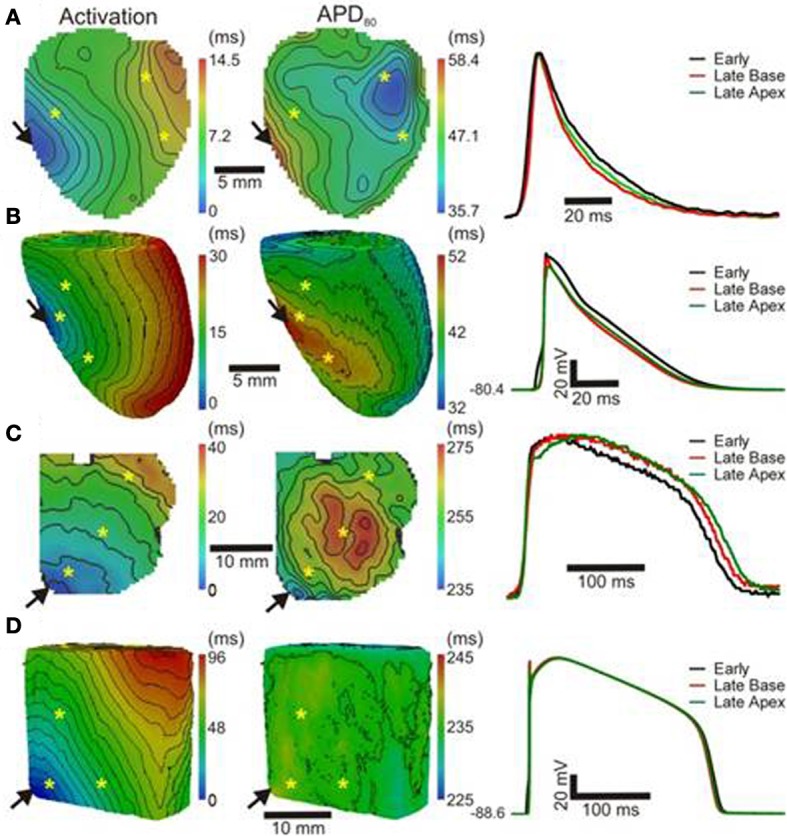
**AT and APD maps from rat and pig myocardium**. Rat ventricles were paced from the anterior insertion sites (arrows) in experiments **(A)** and simulations **(B)**. Pig left ventricular wedge preparations were paced from the apex in experiments **(C)** and simulations **(D)**. Panels (from left to right) are AT, corresponding APD maps and AP traces acquired from pixels indicated by^*^. Isochrones are 1 and 5 ms spacing for rat and pig experiments and 2 and 5 ms for the corresponding simulations. AP traces are aligned by activation times.

Optical images from the epicardial surface of the rat ventricles and the pig left ventricular myocardium were obtained to investigate the relationship between the activation sequence and the spatial distribution of APD_80_. Figure 1 shows activation and APD_80_ maps for epicardial stimulation of the rat and pig tissues at 160 and 496 ms basic cycle lengths, respectively. Figure 1A (left panel) shows the activation map across the rat left ventricle following stimulation at the anterior insertion site (isochrones are shown every 1 ms). A total AT of 14.5 ms was found across the left ventricle. The corresponding spatial distribution of APD_80_ was heterogeneous (middle panel). The longest APD_80_ of 58.4 ms was observed at the pacing location and the shortest of 35.7 ms lay close to the site of the latest AT. The steepest mean spatial gradient of APD (1.6 ± 1.1 ms/mm) was found to lie along the direction of slowest AP propagation. Optical APs along the 10 ms isochrone, one from the base and another from the apex, were compared with an AP from an early (4 ms) activation site and aligned by the maximal derivative of the upstroke (right panel). APs from early activated regions were associated with slowed rates of repolarization relative to APs from late activated regions. Figure 1B shows activation and APD_80_ maps obtained from the simulations using the rat heart model. As in experiments, regions of early activation were associated with the longest APD_80_ (57.7 ms) that became gradually shorter with AT. Spatial gradients of APD_80_ were greatest in the directions of slowest propagation, consistent with experiments. APs extracted from voxels with early and late ATs were aligned by AT and superimposed to illustrate the difference in APD (right). As in experiments, relative prolongation of APDs associated with early ATs could be observed.

Figure 1C (left panel) shows the activation map from a coronary-perfused pig left ventricle that was stimulated from the anterior apical region. Smooth anisotropic propagation was observed across the tissue preparation with a total epicardial AT of 44.4 ms (isochrones are shown every 5 ms). Although a heterogeneous pattern of APD_80_ was found in the pig ventricle (middle panel), dispersion of APD_80_ did not seem to correlate with AT. The longest APD_80_ was observed at the mid-level of the left ventricular wall (276.7 ms) whereas the shortest was found close to the pacing site (237.7 ms). This is further confirmed by examination of individual optical APs obtained at various ATs as shown on the right panel. Interestingly, simulations showed some degree of APD_80_ modulation by the activation sequence with the longest APD_80_ (238.3 ms) near the pacing site (Figure 1D). Yet, this effect was small compared to the simulations and experiments in rat intact hearts. It should be noted that in both rat and pig simulations, base-to-apex gradients of APD were to incorporated in to the ionic models, or moreover a Purkinje fiber network. This will, in part, account for differences in the dispersion of APD and total AT observed between experiments and simulations.

**Figure 2:**
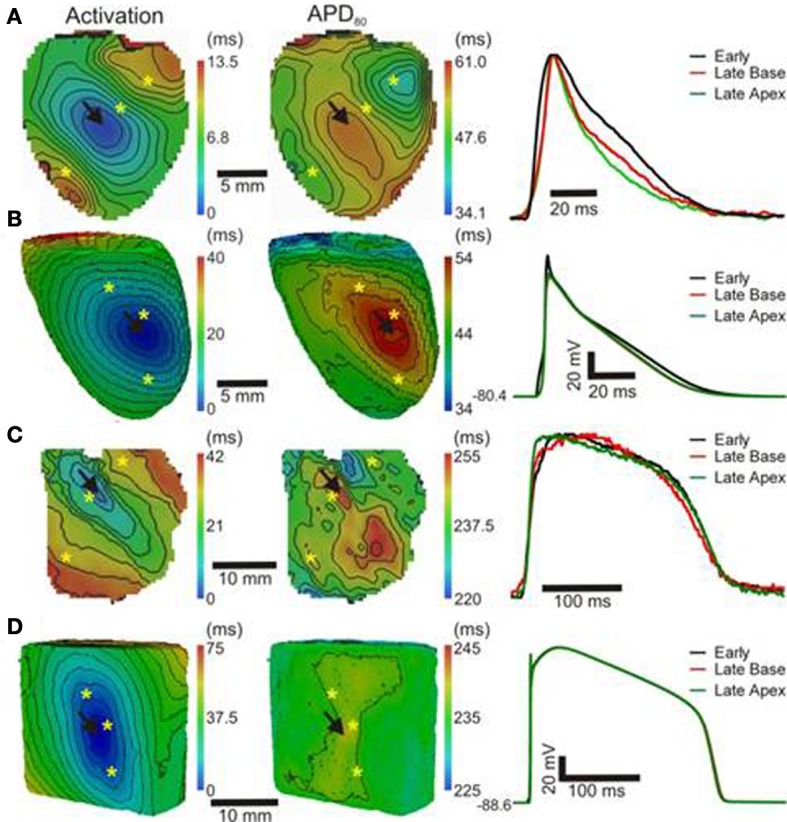
**APD distributions for alternative activation sequences in rat and pig myocardium**. Rat experiments **(A)** and simulations **(B)**; and pig experiments **(C)** and simulations **(D)** with pacing from the mid free left ventricular wall (arrows). Panels (from left to right) are AT, corresponding APD maps and AP traces acquired from pixels indicated by^*^. Isochrones are as in Figure 1. AP traces are aligned by activation times.

In order to further establish the role of activation sequence we investigated APD_80_ distributions from secondary pacing locations in all experiments and simulations. Figure 2 shows activation and APD_80_ maps from the same experiments as in Figure 1 but when pacing the mid-free wall of the left ventricle. Figure 2A indicates that the region of longest APD_80_ in the rat heart was shifted to the new pacing location (compare with Figure 1A). APD_80_ became progressively shorter away from this site, predominantly in the direction of slow conduction (optical APs shown in right panel). A shift in APD_80_ to the site of earliest activation was consistently observed in simulations (Figure 2B). Conversely, changing the pacing location in the pig myocardium did not significantly alter the distribution of APD_80_ in experiments (Figure 2C). Although a region of long APD_80_ was evident at the pacing location in simulations (Figure 2D), the spatial gradient of APD_80_ across the epicardial surface was very shallow relative to that observed in the rat.

### Relationship between action potential duration and the activation sequence

**Figure 3:**
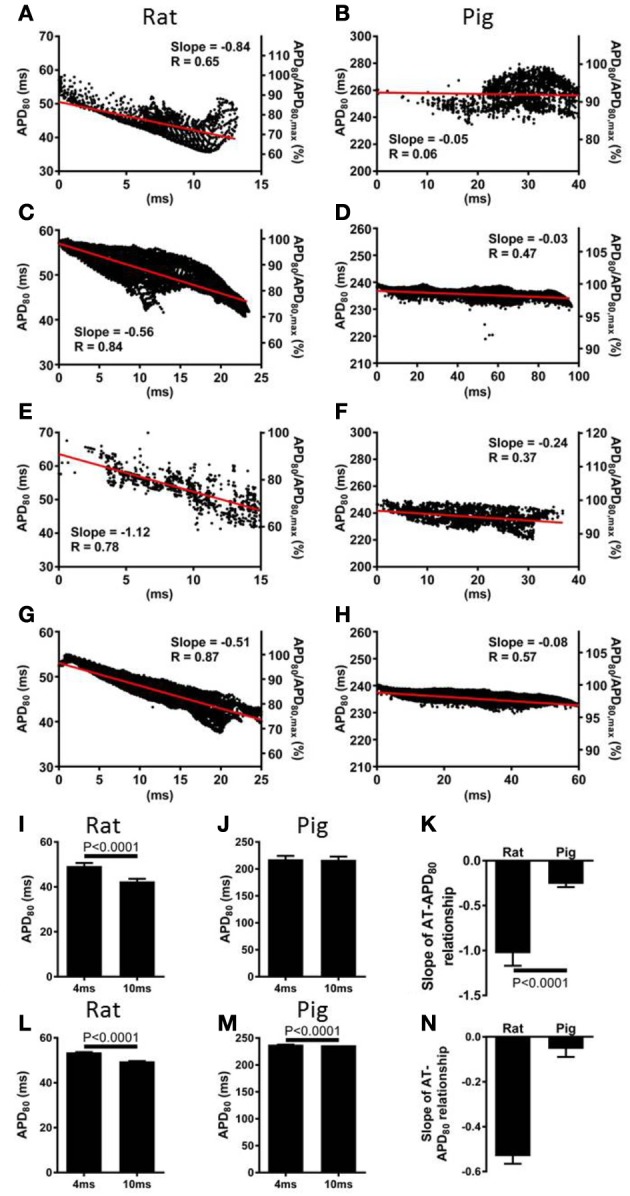
**Correlations between AT and APD in rat and pig myocardium**. AT and APD plots corresponding to maps shown in Figure 1 for rat **(A)** and pig **(B)** experiments. Slopes for linear correlation analyses are indicated. AT and APD plots of corresponding simulations are shown for rat **(C)** and pig **(D)**. Similarly, AT and APD plots from an alternative pacing location, as in Figure 2, are shown for rat **(E)** and pig **(F)** experiments and rat **(G)** and pig **(H)** simulations. Panels **(A–H)** are also expressed as a percentage of the maximum APD_80_. Mean ± *SD* APD corresponding to 4 and 10 ms activation isochrones across all experiments and pooled from each pacing location in rat **(I)** and pig **(J)**. **(K)** Equally, slopes of linear correlations between AT and APD were quantified for all experiments and pacing locations and expressed as Mean ± *SD*. Similarly to experiments, early vs. late activation times were compared for rat **(L)** and pig **(M)** simulations shown in Figure 2. **(N)** Slopes from linear correlations of corresponding AT and APD data are compared. All mean differences were compared using *t*-tests. Significant differences are indicated if *P* < 0.05.

The relationship between AT and APD_80_ at each pixel of the imaged surface was quantified by linear regression analysis. Representative data for rat and pig experiments are shown in Figures 3A,B, respectively. For all AT-APD plots, the relative APD_80_ from the maximum APD_80_ is shown by the secondary *y*-axes. These plots correspond to the activation and APD_80_ maps shown in Figure 1 whereby tissue was paced from the mid-anterior insertion site in rat and apical region of LV in pig. A clear decreasing trend of APD_80_ with AT was revealed in the rat (slope = −0.84, *R* = 0.65) compared with a relatively shallow relationship and poor correlation in the pig wedge preparation (slope = −0.05, *R* = 0.06). Mean ± *SD* of *R* values of linear regression were significantly less in pig than rat (0.25 ± 0.22 vs. 0.66 ± 0.18, *p* = 0.0002). Relative to the maximum APD_80_ observed on the imaged surface, APD_80_ was decreased by as much as 38.9% in rat but only 16.0% in pig. Linear regression analyses of AT and APD_80_ relationships in rat (Figure 3C) and pig (Figure 3D) simulations were qualitatively similar to experiments. We further show that upon changing the location of pacing of the tissue to the mid-LV free wall, the steep AT, and APD relationship of rat simulations (Figure 3E) and shallow relationship of pig simulations (Figure 3F) were conserved. This data is further supported by statistical comparison of APD_80_ from early (4 ms) and late (10 ms) ATs. Across all rat hearts and stimulation sites (Figure 3I), a significant decrease in APD_80_ (13.8%) was found between early and late activated zones (*p* < 0.0001), while no significant difference was observed in the pig wedge (Figure 3J). Correlations between AT and APD_80_ for rat and pig experiments were further assessed by comparison between slopes of the linear relationship between APD_80_ and AT. Figure 3K shows that AT-APD_80_ relationships across all experiments were significantly steeper in rats compared to pigs (*p* < 0.0001). In simulations, AT and APD_80_ values were extracted from the epicardial surface of the LV free wall in accordance with the imaging plane of experiments. We found a significant decrease in APD with AT in both the rat and pig model. However, over a 6 ms interval in AT when pacing the mid-LV fee wall, APD decreased by 7.57% in rat and by only 0.4% in pig (Figures 3L,M). The slope of AT and APD_80_ relationship in pig was only 11.6% of that observed in rat (Figure 3N). For comparison, we report that when pacing the mid-anterior insertion site in rat and apical region of the LV in pig, similar differences of Mean ± *SD* APD_80_ between 4 and 10 ms isochrones were identified as for the aforementioned pacing location (7.1 and 0.3%). Accordingly, a difference in the slope of AT and APD_80_ relationships between rat and pig was observed (29.6%).

### The dispersion of repolarization time

**Figure 4:**
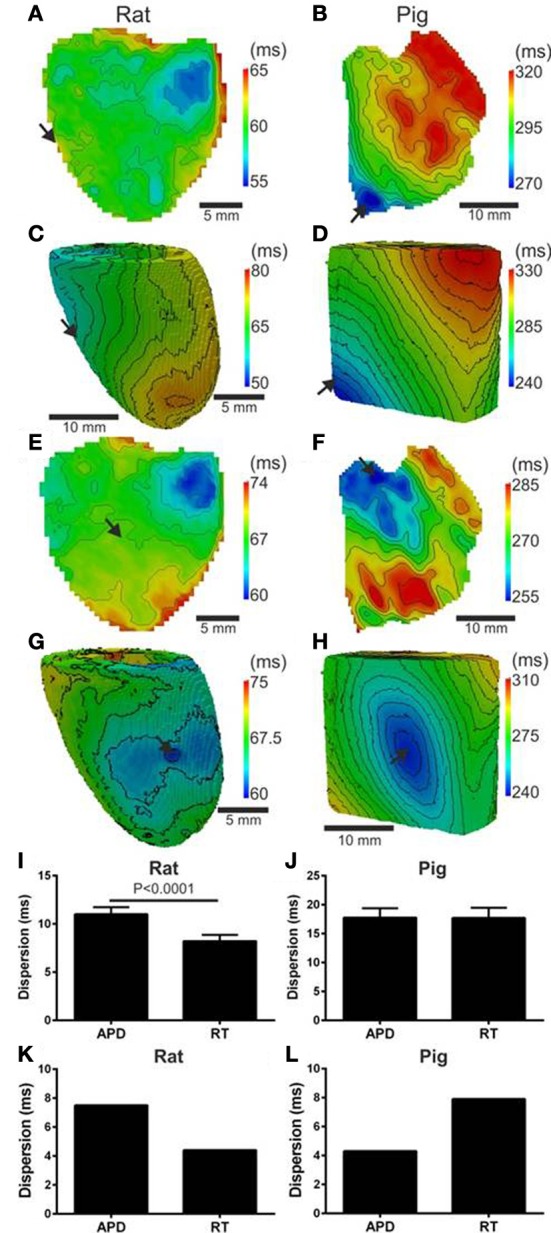
**Left ventricular dispersion of RT in rat and pig myocardium**. RT maps corresponding to maps shown in Figure 1 for rat **(A)** and pig **(B)** experiments and rat **(C)** and pig **(D)** simulations. Similarly, RT maps for an alternative pacing location, as in Figure 2, are shown for rat **(E)** and pig **(F)** and repeated for rat **(G)** and pig **(H)** simulations. Stimulation sites are indicated by arrows. Mean ± *SD* dispersion of APD and RT pooled from each pacing location are compared across all experiments in rat **(I)** and pig **(J)**. All mean differences from experiments were compared using paired *t*-tests. Significant differences are indicated if *P* < 0.05. Similarly to experiments, dispersion of APD and RT were compared for rat **(K)** and pig **(L)** simulations shown in Figure 2.

Since local RT depends on local APD, it is to be expected that modulation of APD by the activation sequence affects RT dispersion. Figure 4A shows that, in rat, progressive shortening of the APD throughout the activation sequence plays an important role in homogenizing the spatial dispersion of RT. However, for the pig, a lack of association between APD and AT resulted in RT to follow the sequence of activation and intrinsic heterogeneities of APD (Figure 4B). As total AT of the rat LV is typically <15 ms, the dispersion of RT was calculated from pixels corresponding to the earliest 10 ms of AT. The dispersion of RT (RT_95%_–RT_5%_) was 7 and 14 ms for the rat and pig shown in Figures 4A,B, respectively. Simulations were in accordance with experiments as seen by relatively homogeneous epicardial RT maps in rat whereas the reverse was seen for pig (Figures 4C,D). Similarly using the secondary pacing location, RT was relatively homogeneous in rat compared to pig. The dispersion of RT was seemingly consistent in rat at 8 (Figure 4E) and 16 ms in pig (Figure 4F). Simulations of the secondary pacing locations were again consistent with experiments (Figures 4G,H).

Comparisons of Mean ± *SD* (pooled from each pacing location) APD_80_ dispersion and RT dispersion for rat and pig experiments are shown in Figures 4I,J. The dispersion of RT is less than APD_80_ by 25.3% (*p* < 0.0001) in rat (Figure 4I) whereas no significant difference was observed in pig (Figure 4J). In simulations pacing at the mid-LV free wall the percentage change of APD_80_ dispersion to RT dispersion supported experimental findings showing reduced RT dispersion in rat (41.3%, Figure 4K) but a relative and robust gain of RT dispersion in pig (83.7%, Figure 4L).

### Modulation of action potential duration and repolarization time by tissue architecture

**Figure 5:**
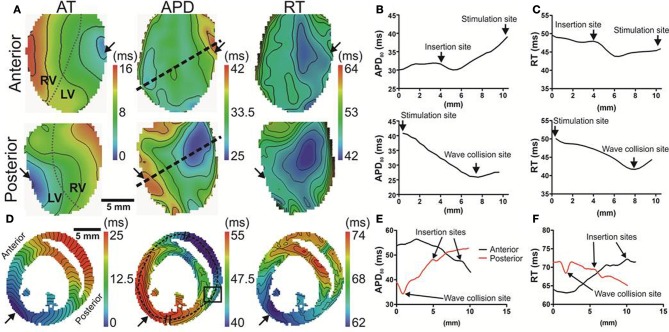
**Local modulation of APD in rat hearts by tissue architecture. (A)** Hearts were simultaneously imaged from anterior (upper panels) and posterior (lower panels) views. Panels (from left to right) are AT and APD maps from ventricles paced at the mid left ventricular free wall (arrows). Insertion sites of left and right ventricular free walls are indicated by dotted lines in AT maps. Isochrones are spaced 2 ms. Linear profiles of APD **(B)** and RT **(C)** measured from anterior (upper panel) and posterior (lower panel) surfaces. APD values were taken along linear profiles intersecting the longest and shortest APD from each imaged surface (dashed lines in Panel **A**). Regions corresponding to pacing sites, insertion of ventricles and convergence of anterior and posterior wave fronts are indicated. **(D)** AT and APD maps in simulations are shown in the transverse plane. Isochrones are spaced 1 ms apart. A region of long APD was identified at the insertion site (box). Transmural profiles of APD **(E)** and RT **(F)** are plotted from pixels along dashed lines shown in **(D)**.

In our rat heart experiments we found that the insertion sites of the two ventricles were the only regions where the AT-APD_80_ relationship could significantly be altered. Figure 5A shows a wave front propagating from a left ventricular mid-free wall pacing site to the right ventricle (left panels). In these experiments anterior and posterior activity was acquired simultaneously. The corresponding APD_80_ and RT distributions are shown in the middle and right panels, respectively. The longest and shortest APD_80_ were still located at the pacing site and the region of latest activation, respectively. Figure 5B shows APD_80_ profiles obtained from the maps in Figure 5A (dashed line). The profile was chosen through the pacing location and the site of shortest APD_80_. On the anterior side, a region coinciding with the boundary between the left and right ventricle showed a local maximum in APD_80_ (31.9 ms). This had a significant effect on the local RT with a local increase of as much as 4.1 ms (Figure 5C). Computer simulations in the 3D model of the rat ventricle allow investigation of this phenomenon in more detail. Figure 5D shows transmural activation and APD_80_ maps in the model. The AP propagated anisotropically from the left ventricle into the septum and right free wall (left panel). The APD_80_ map reveals a region of enhanced APD_80_, and consequently RT, localized to the septal-ventricular branching site (black box). Furthermore, the site of wave front collisions in the RV and septum were associated with significantly decreased APD_80_, RT and enhanced mean gradients of APD_80_ shortening compared to those observed in the LV free wall (1.4 vs. 0.2 ms/mm). Figures 5E,F further illustrates these findings by plotting APD_80_ and RT, respectively, along the transmural profiles indicated in Figure 5C (dashed line).

### Rate-dependency of electrotonic modulation of repolarization

**Figure 6:**
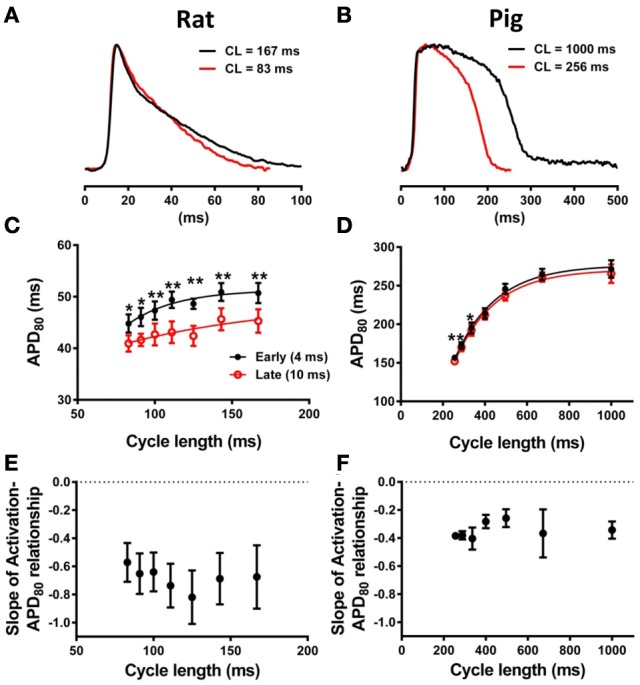
**Restitution properties of APD_80_ and its dependence on AT. (A)** Representative optical AP traces extracted from the same pixel location during pacing with cycle lengths, CL, of 167 and 83ms in rat. **(B)** Similarly, optical AP traces from pig paced at 1000 and 256 ms cycle lengths are superimposed. Mean ± *SD* APD_80_ from 4 and 10 ms isochrones were plotted against pacing cycle length for rat **(C)** and pig **(D)** (*N* = 7 and *N* = 5, respectively). Statistical differences between APD across all pacing frequencies were determined by paired *t*-tests (^*^P < 0.05; ^**^P < 0.01). For comparison of the steepness of restitution, data were fitted with one-phase decay exponential curves. Mean ± *SD* of slopes of linear relationships between AT and APD_80_ were plotted against pacing cycle length for rat **(E)** and pig **(F)**. One-way ANOVA was used to determine statistical data variance across pacing cycle lengths tested (*P* < 0.05). No statistical deviations between means of slopes were observed for rat or pig.

In our experiments a strong relationship between APD_80_ and AT was preserved at different pacing frequencies in rat but not in pig. APs from short and long pacing cycle lengths are shown for rat (Figure 6A) and pig (Figure 6B) experiments. Figure 6C shows APD_80_ restitution curves from locations corresponding to relatively early (4 ms) and relatively late AT (10 ms) isochrones. Throughout the range of pacing cycle lengths applied in rat, ranging from 83 to 167 ms, the largest mean difference in APD_80_ between early and late AT data were found at longer pacing cycle lengths. At shorter pacing cycle lengths restitution curves tended toward convergence as indicated by a reduction in statistical power (although still significant). Convergence of APD_80_ restitution data at short cycle lengths was attributed to variation in the maximum slope of the restitution profile between early and late regions of AT. The maximal slope of APD_80_ data restitution was larger at regions of early AT compared with late AT (0.21 vs. 0.08). In pig experiments, APD_80_ restitution was largely unaffected by AT between pacing cycle lengths ranging from 256 to 1000 ms (Figure 6D). Significant differences of APD_80_ were observed between early and late AT at 288 and 336 ms only. Despite modest differences in mean APD_80_ at specific cycle lengths, the maximal slope of restitution was equal (0.63) between early and late AT.

To determine the rate-dependency of electrotonic modulation of repolarization, the slope of linear correlations of the AT-APD_80_ relationship were quantified across all pacing cycle lengths in rat and pig experiments. Figure 6E shows the lack of dependence of the AT-APD_80_ slope on pacing cycle length. The mean of the slope was preserved between a range of −0.57 and −0.82. One-way analysis of variance determined no statistical variation within the sampled pacing cycle lengths (*P* > 0.05). Similarly for pig, slopes of AT-APD_80_ relationships were unaffected by pacing cycle length (Figure 6F). However, slopes were consistently less steep than for rat, ranging from −0.25 to −0.4 with no statistically significant variation for different pacing cycle lengths (*P* > 0.05).

### Determinants of electrotonic load

**Figure 7:**
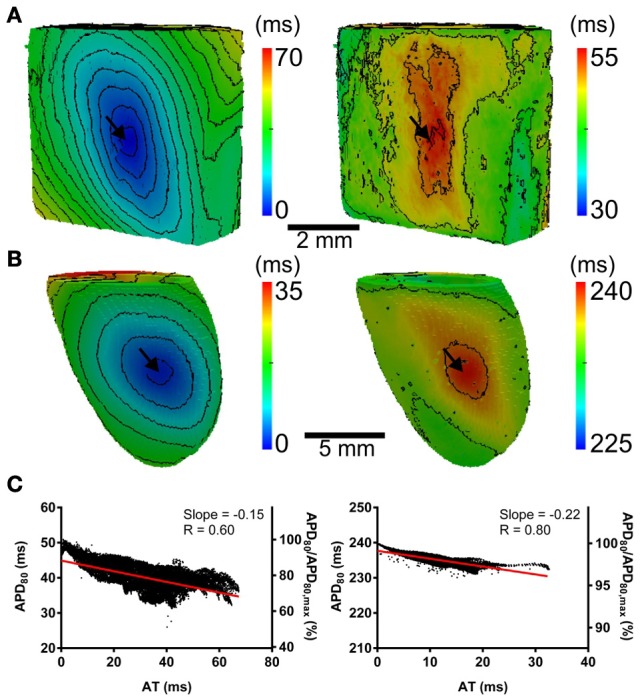
**The influence of electrophysiological kinetics and tissue geometry on the dispersion of APD**. Electrophysiological kinetics for rat (R_k_) and pig (P_k_) simulations was interchanged between geometries (R_g_ and P_g_, respectively). Ventricles were paced at the mid LV free wall (arrows). Panels are maps of AT (left panels) and APD_80_ (right panels) derived from simulations in the following configurations: R_k_P_g_ **(A)** P_k_R_g_ **(B)**. Isochrones are 4ms for all maps. **(C)** AT-APD plots for R_k_P_g_ (left panel) and P_k_R_g_ (right panel) simulations. Slopes of linear correlation analyses are indicated. Secondary *y*-axes show APD_80_ expressed as a percentage of maximum APD_80_.

**Figure 8:**
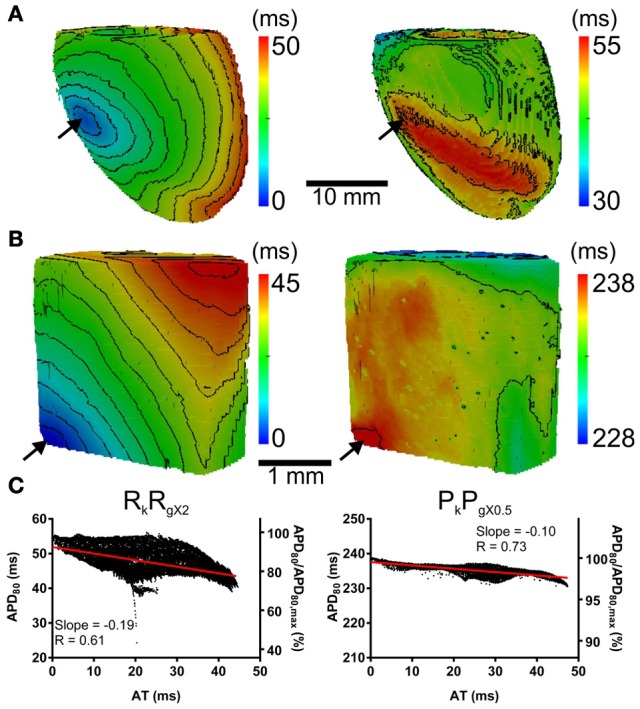
**The influence of tissue geometry on the dispersion of APD**. Ventricles were paced at the mid-anterior insertion site of rat and apex of pig geometries (arrows). Panels are maps of AT (left panels) and APD (right panels). **(A)** Dimensions of rat geometries were increased by a factor of two (R_k_R_gX2_). **(B)** Pig kinetics were simulated in a pig geometry with dimensions scaled by half (P_k_P_gX0.5_). Isochrones are 4 ms for all maps. **(C)** AT-APD plots for R_k_R_gX2_ (left panel) and P_k_P_gX0.5_ (right panel) simulations. Slopes of linear correlation analyses are indicated. Secondary *y*-axes show APD_80_ expressed as a percentage of maximum APD_80_.

**Figure 9:**
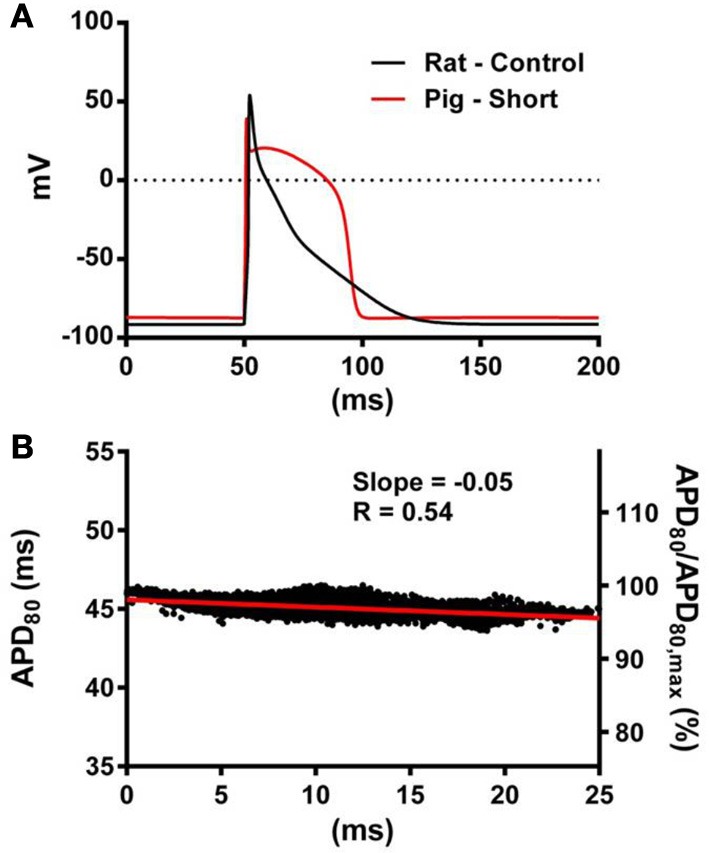
**The influence of AP morphology on coupling of AT and APD**. In the rat geometry simulations of rat kinetics were compared to a modified pig kinetics model (P_k,short_R_g_) whereby *G*_Ks_ and *G*_Ks_ were increased by a factor of 28.5 to match the APD_80_ of the rat model. **(A)** Single cell AP traces comparing the rat and modified pig kinetics. Whole rat ventricle simulations were paced at the mid LV free wall. **(B)** AT-APD plot shown for P_k,short_R_g_ simulation. The comparative AT-APD plot for an R_k_R_g_ simulation is shown in Figure 3G. Slope of linear correlation analyses is indicated. Secondary *y*-axes show APD_80_ expressed as a percentage of maximum APD_80_.

The computational models allow investigation of the relative roles of AP shape (or kinetics), tissue size and tissue geometry on the electrotonic modulation of repolarization. This was first assessed by interchanging rat and pig AP kinetics (R_k_ and P_k_, respectively) from simulations in rat and pig tissue geometries (R_g_ and P_g_, respectively). In Figure 7 R_k_P_g_ and P_k_R_g_ simulations were paced at the mid LV free wall, consistent with Figure 2. Figures 7A,B show AT and APD_80_ maps for the R_k_P_g_ and P_k_R_g_ simulations, respectively. The regions of longest APD_80_ was observed close to sites of stimulation in each simulation. In Figure 7C APD_80_ is plotted against AT for the R_k_P_g_ (left panel) and P_k_R_g_ (right panel) simulations. Maximal spatial in plane dispersion of APD_80_ was as much as 48.9% of the maximal epicardial APD_80_ in R_k_P_g_ simulations, but only 4.1% in P_k_R_g_. The slope of the AT-APD_80_ relationship became less negative by 54.9 and 58.6% when comparing R_k_R_g_ to P_k_R_g_ and R_k_P_g_, respectively. Conversely, the steepness of this relationship became more pronounced by 66.3and 52.5% when comparing P_k_P_g_ with P_k_R_g_ and R_k_P_g_. The computational models were further used to assess the effects of tissue size alone on electrotonic modulation of repolarization by either increasing or decreasing the tissue dimensions by a factor of two in the rat geometry (R_gx2_) (Figure 8A) and pig wedge (P_gx0.5_) (Figure 8B). In Figure 8C APD_80_ is plotted against AT for R_k_R_gx2_ and P_k_P_gx0.5_ simulations, respectively. We find that by comparison to Figure 3, the slope of the AT-APD_80_ relationship becomes less negative by enlargement of rat heart simulations (−0.19 vs. −0.56). The converse was observed in the pig wedge of reduced dimensions, where an increased association between AT and APD_80_ is observed relative to simulations of true dimensions (−0.1 vs. −0.03 AT-APD_80_ slope). To ascertain the effects of AP morphology alone on electrotonic modulation of repolarization, we compared a modified pig kinetics model with an equivalent APD_80_ to the rat model. Figure 9A shows that the modified pig model retained a spike and dome-like AP with a considerably shortened plateau phase when compared to the unmodified pig AP (Figures 1, 2) and an increased rate of repolarization during phase 3, relative to the rat AP. Despite APD_80_ being similar, epicardial APD_80_ in the P_k,short_R_g_ simulation showed little dependence on AT (Figure 9B). The slope of AT-APD relationship was less by as much as 92.0% compared to R_k_R_g_ simulation (shown in Figure 3G).

**Figure 10:**
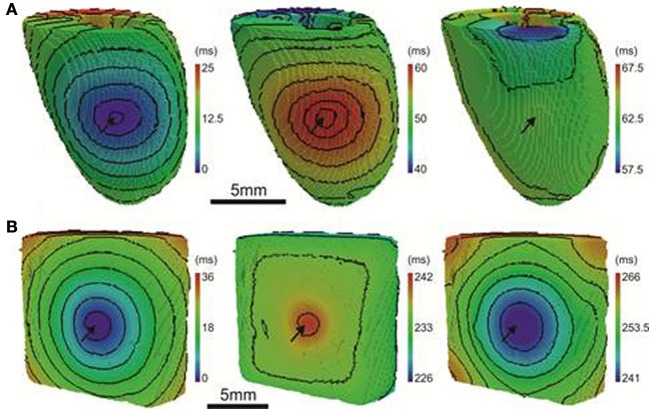
**AT, APD, and RT maps for isotropic simulations**. In rat **(A)** and pig **(B)** simulations, the activation sequences (left panels) show no preferential direction of propagation. Sites of stimulation are indicated by arrows. The distribution of APD (middle panels) is tightly coupled to the activation sequence in rat simulations but to a much lesser extent in the pig. A decreasing trend of APD away from the pacing site was observed uniformly in all directions across the epicardial surface. A similar pattern of APD was observed in the pig simulations however the range of APD values were much smaller. The RT map was relatively homogeneous in the rat simulations compared to the pig (right panels). The RT pattern in pig closely followed the activation sequence. Isochrones are spaced 2 and 4 ms apart for rat and pig, respectively.

**Figure 11:**
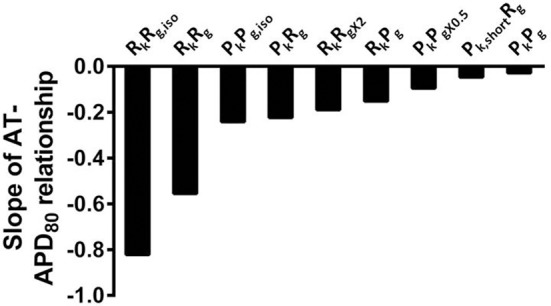
**The influence of anisotropy, AP morphology, and tissue geometry on the correlation between activation and APD in simulations**. Comparison between slopes of linear correlations for each simulation in Figures 2, 7–10.

For comparison to anisotropic simulations, isotropic (no fibers) simulations in rat heart and pig wedge geometries were performed. Here the uniform spread of excitation in rat was associated with near uniform gradients of APD_80_ away from the site of stimulus (Figure 10A). APD_80_ was found to change near 1:1 with AT shown by a change of 6 ms of AT, from 4 to 10 ms isochrones, modulated APD_80_ from a Mean ± *SD* of 57.5 ± 0.5 ms to 52.5 ± 1.3 ms. Therefore, a near simultaneous repolarization time was observed in the left ventricle of the isotropic rat simulation. Simulations in isotropic wedges also revealed a strengthening, although modest, of the relationship between AT and APD using the pig model compared to anisotropic wedges (Figure 10B). Despite this, early vs. late AT modulated APD_80_ from a Mean ± *SD* of 238.9 ± 0.3 ms to only 237.1 ± 0.3 ms. Consequently, significant dispersion of RT was still observed, and followed the activation sequence. Figure 11 summarizes the slopes from linear correlations of APD_80_ plotted against AT for the simulations in Figures 2, 7–10.

### Transmural dispersion of action potential duration

**Figure 12:**
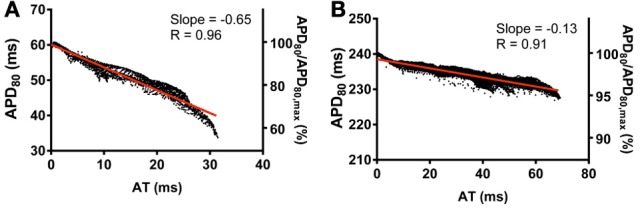
**Correlations between AT and APD in the transmural plane of rat (A) and pig (B) myocardium**. AT and APD_80_ plots corresponding to the horizontal transmural plane at the level of the pacing site in simulations from Figure 2. Slopes for linear correlation analyses are indicated. Secondary *y*-axes show APD_80_ expressed as a percentage of maximum APD_80_.

Finally, the influence of tissue geometry in the transmural plane on electrotonic modulation of repolarization was investigated. AT and APD_80_ values were extracted from transmural slices corresponding to the level of the pacing site for both rat (R_k_R_g_) and pig (P_k_P_g_) models. AT-APD_80_ relationships for rat and pig revealed clear descending linear distributions of APD_80_ with AT (Figures 12A,B, respectively). For the R_k_R_g_ simulation, the shortest APD_80_ was observed at the latest AT with an APD_80_ that was 44.6% shorter than the maximal APD_80_ within the transmural plane. Percentage shortening of APD_80_ in P_k_P_g_ was substantially less at 5.6%. The steepness of these relationships was −0.65 and −0.13 for rat and pig, which corresponds to increases of 27.5 and 62.5% in the transmural plane compared to the epicardial relationships, respectively. Similarly, AT-APD_80_ relationships were associated with augmented *R* values in both rat (0.96 vs. 0.87) and pig (0.91 vs. 0.57) models.

## Discussion

### General discussion

The focus of the present study was to characterize the dynamic interplay between APD and the activation sequence in the myocardium with an aim to identify the mechanisms underlying acute electrotonic modulation of repolarization. Such mechanisms were investigated experimentally and computationally by comparing two species, rat, and pig, with distinct electrophysiological properties and two different tissue geometries, the whole heart and the ventricular slab. Using epi-fluorescence optical imaging we found a strong relationship between APD and AT in healthy rat hearts, leading to a large dispersion of APD, but relatively homogeneous RTs. This effect was especially pronounced in directions of slow conduction (Figures 1, 2, 4). In pig left ventricular wedge preparations however, spatial distributions of APD showed a relatively poor dependence on the activation sequence, consistent with previous studies (Lacroix et al., 1999; Kongstad et al., 2002; Liang et al., 2005). These results were consistent from two different pacing locations tested in each experimental model and simulations.

Our experimental results are in agreement with earlier computational studies that predicted an important role for the shape of the cardiac AP (Sampson and Henriquez, 2005; Cherry and Fenton, 2011). The electrotonic current is proportional to the membrane potential difference between neighboring cells and depends on the dynamic membrane resistance during repolarization (Sampson and Henriquez, 2005). In pig, due to the plateau phase, regions of tissue activated early in the activation sequence remain in relatively depolarized states long after all tissue has been activated. This significantly reduces the overall spatial gradient of the transmembrane potential during repolarization and consequently dampens its electrotonic modulation. Furthermore, the late phase 3 repolarization is strongly dominated by outward potassium currents and relatively small membrane resistance and therefore less sensitive to external currents. Conversely, the rat AP shape provides relatively constant membrane potential gradients throughout the whole repolarization duration. The late repolarization phase in the rat heart is also occurring at relatively slow rates, indicating a delicate balance between outward and inward currents, and is thus more susceptible to be affected by electrotonic currents (Vigmond et al., 2009). Our study is the first to investigate in detail and highlight the differences in electrotonic modulation of repolarization between two different mammalian species experimentally.

The rate dependency of the AT-APD relationship was investigated experimentally in both rat and pig ventricles. Reducing the pacing cycle length of tissue leads to AP shortening in both rat and pig myocardium. In our experiments, the shape of the AP, in terms of the rate of phase 3 repolarization, was largely preserved across all pacing cycle lengths for each species, as can be observed in Figures 6A,B. We did not observe a significant deviation of the slope of AT-APD relationship across pacing cycle lengths in either rat or pig experiments. Thus, it appears that irrespective of APD and pacing cycle length, the shape of the AP (triangular vs. spike-and-dome) is a main determinant of electrotonic modulation of repolarization. This is further supported by simulations shown in Figure 9 that showed that shortening the pig APD (by abbreviation of the plateau phase whilst retaining a spike and dome AP) to match the rat APD, did not improve the relationship between AT and APD_80_. These results show clearly that AP morphology plays a crucial role in electrotonic modulation of repolarization whereby the triangulated AP of the rat has significantly greater coupling between AT and APD than a spike-and-dome AP, as in pig. Hanson et al. (2009) using, S1S2 stimulation from the endocardium in human ventricles, observed that AT and activation-recovery interval (analogous to APD) reduced the steepness of linear correlations at S1S2 coupling intervals close to the shortest effective coupling interval. This study utilized noncontact balloon electrodes and therefore could not report on the AP morphology. However, S1S2 stimulation protocol may impose differential effects on AP morphology compared with dynamic stimulation since it is well established that the maximal slope of APD restitution is significantly enhanced by the S1S2 pacing protocol (Osadchii, 2012).

The role of tissue geometry on electrotonic modulation of repolarization has previously been investigated in theoretical models of cardiac propagation. Wang and Rudy (2000) used simulations of single ventricular muscle fibers that expand to multiple fibers, thus enhancing the number of neighboring cells at the site of the expansion, and found localized changes to APD at the sites of branching due to enhanced electrotonic load from fiber branches. More recently, Cherry and Fenton (2011) predicted a similar effect occurring at the insertions site of the left and right ventricle, and the septum, in homogeneous but anisotropic two-dimensional simulations of an axial slice of dog heart. In the present study, we investigated experimentally the relative role of tissue geometry on the electrotonic modulation of repolarization. We showed, for the first time, that the effect previously proposed by Cherry and Fenton (2011) was present at the level of the whole heart, both in experiments and simulations. Activation from the left ventricular free wall toward the right ventricle in the rat heart was associated with decreasing APD in a near linear trend with the exception of the border region between left and right ventricles (Figure 5). A band, extending from the base to the apex, of prolonged APDs was observed. This observation was replicated in 3D simulations using the rat geometry. Here, a localized region of prolonged APD_80_ was observed throughout the ventricular wall at the intersection of the septum and right ventricular free wall with the left ventricle. Furthermore, this effect significantly altered the intrinsic transmural APD gradient that was implemented in the cellular model (Pandit et al., 2001).

Computational models are a useful tool for investigating the relative role of AP kinetics and tissue properties in the electrotonic modulation of repolarization. In our study we ran additional simulations whereby the AP kinetics of one species was incorporated into the geometry of the other, and vice versa (Figures 7, 11). When using the rat AP and changing the geometry from the rat whole ventricular model to the pig wedge preparation, we observed a pronounced reduction in the steepness of the AT-APD_80_ relationship. However, when using pig AP kinetics in the rat whole ventricular model we found a more pronounced correlation between AT and APD_80_ than when using the pig wedge geometry. We also found that scaling up the rat geometry reduced the steepness of the AT-APD_80_ relationship (Figure 8). Reducing the size of the pig geometry increased the steepness of the linear AT-APD_80_ relationship. Furthermore, uniform activation sequences in isotropic simulations were accompanied by tighter coupling of APD_80_ to AT as shown by more pronounced negative slopes of linear regression analysis compared to anisotropic simulations for both, rat and pig (Figure 11). The magnitude of the slope for isotropic simulations was still substantially greater in rat than in pig. In addition, the AT-APD_80_ relationship of the transmural plane was found to be steeper than on the epicardium (Figure 12). These results are consistent with previous two-dimensional simulation studies that showed that electrotonic APD modulation is strongest at pacing sites, tissue boundaries, and wave collisions sites. Increasing tissue size effectively reduces the contributions of the pacing site and tissue boundaries to the overall AT-APD_80_ relationship, thus leading to less pronounced correlations and slopes. These results clearly demonstrate that geometry and tissue size play an important role in determining spatial gradients of APD_80_ in tissue.

Dispersion of RT is an important determinant of the vulnerability to arrhythmias following a premature activation. In our experiments we found that, due to electrotonic interactions, RT was relatively homogeneous in the paced intact rat heart, but more heterogeneous in the pig ventricle (Figure 4). These results suggest that the rat heart would be more resistant against arrhythmias following ectopic activation, than pig ventricles. Electrotonic modulation of repolarization could therefore be a contributing factor in the reduced occurrence of sudden cardiac deaths in murine animal models compared to larger species (Sabir et al., 2008). However, our study has also highlighted structural features that could provide pro-arrhythmic substrates even in the smaller hearts: regions of tissue expansion at the insertion site between left and right ventricles were associated with APD prolongation and increased RT dispersion (Zubair et al., 1994). Therefore, regions of expanding conductive tissues such as ventricular insertion sites, insertion of papillary muscles and trabeculae carneae, could have increased vulnerability to conduction block following premature stimuli. This study provides the first experimental evidence supporting a mechanism previously only demonstrated computationally (Cherry and Fenton, 2011) for arrhythmogenesis underlayed by a structural substrate.

### Future directions

The present study has shown the influence of electrotonic effects for differing tissue structures and AP morphologies of the rat and pig ventricles. Future studies will be aimed at investigating in more detail the relative influence of intercellular coupling and membrane resistance in different species. Equally, it would be important to investigate this phenomenon in cardiac diseases.

### Limitations

All optical mapping recordings in the current study were obtained from the epicardium. Transmural electrophysiological heterogeneities are well documented at a cellular level in various species, but to what extent these are modulated by electrotonic currents in tissues remains a topic of debate (Antzelevitch et al., 1998). Fully depth-resolved transmural imaging of APs is currently not feasible, thus making it difficult to directly address this important question. Nevertheless, our computer simulations indicate that electrotonic modulation by the activation sequence is stronger in the transmural direction than epicardially, especially in thin ventricles (see Figures 5, 12), which can be sufficient to overcome intrinsic transmural heterogeneities. These findings are in accordance with previous computational studies (Sampson and Henriquez, 2005; Walton et al., 2010) and corroborate experimental findings on the rabbit left ventricular wedge cut surface (Myles et al., 2010) and in the intact rat heart (Walton et al., 2010). Furthermore, non-uniformities in tissue geometry can greatly affect transmural patterns of repolarization (Figure 5). Electrotonic modulation by the activation sequence of transmural repolarization dispersion is therefore likely to be important, even in the large intact mammalian heart.

We used an electro-mechanical uncoupler in the experiments to prevent motion artifacts and reliably measure repolarization optically. Furthermore, all computational simulations ignored cardiac contraction and electro-mechanical coupling. It is well-known that stretch can affect repolarization (Kohl et al., 2011). Activation of the ventricles from an electrode or ectopic source will create heterogeneous stretch patterns during repolarization that are likely to impact on dispersion of repolarization. To what extent stretch will impact on the findings of the current study remains to be investigated, however a tight coupling between activation and repolarization has been reported in several *in vivo* studies (Hanson et al., 2009; Yue et al., 2005).

There are differences in absolute AT between simulations and experimental data in our study.

Although realistic geometries are used, several factors may account for differences in total AT between simulations and experiments: (1) we have used a single geometry for the rat heart and pig wedge obtained from separate DT-MRI experiments. Therefore, variations in tissue size and electrophysiological properties across the various optical mapping experiments are not accounted for in simulations; (2) the simulations are run on the complete tissue sample (whole heart or wedge) whereas optical mapping data is obtained from a limited field-of-view; (3) transmural electrophysiological heterogeneities were included based on available experimental data. However, no other heterogeneities were incorporated in our models (such as base-to-apex gradients or differential connexin expression) due to the lack of established data; (4) retrograde activation through the Purkinje network may contribute to faster activation in experiment vs. simulation. However, our simulations are in excellent qualitative agreement with experimental data in terms of electrotonic modulation of repolarization.

In our simulations we used a monodomain approach without taking the tissue bath into account. However, this bath is known to increase electrotonic load at the tissue boundaries or at tissue heterogeneities such as vessels [see for example (Bishop et al., 2011; Kelly et al., 2013)]. Bath-loading at the tissue boundary is therefore likely to enhance the boundary effects we observe in our monodomain simulations and could play a role in further modulating the transmural APD gradient. The main conclusions of our study remain however valid.

## Conclusion

In conclusion, our study has highlighted that electrophysiological and structural differences between species and the type of tissue preparations have significant roles in electrotonic modulation of repolarization. AP shape, tissue size and tissue geometry should all be taken into account when investigating dispersion of repolarization in cardiac tissue.

### Conflict of interest statement

The authors declare that the research was conducted in the absence of any commercial or financial relationships that could be construed as a potential conflict of interest.

## Abbreviations

3D: Three-dimensional
AP: Action potential
APD: Action potential duration
APD_80_: Action potential duration at 80% of repolarization
AT: Activation Time
DT-MRI: Diffusion Tensor Magnetic Resonance Imaging
LV: Left ventricle
P_g_: Pig geometry
P_k_: Pig kinetics
R_g_: Rat geometry
R_k_: Rat kinetics
RT: Repolarization time
RT_max_: Maximum repolarization time
RT_min_: Minimum repolarization time.
